# Heterogeneous glycosylation and methylation of the *Aeromonas caviae* flagellin

**DOI:** 10.1002/mbo3.1306

**Published:** 2022-07-18

**Authors:** Rebecca C. Lowry, Laila Allihaybi, Jennifer L. Parker, Narciso A. S. Couto, Graham P. Stafford, Jonathan G. Shaw

**Affiliations:** ^1^ Department of Infection Immunity and Cardiovascular Disease University of Sheffield Medical School Sheffield UK; ^2^ Chemical and Biological Engineering University of Sheffield Sheffield UK; ^3^ School of Clinical Dentistry, Claremont Crescent University of Sheffield Sheffield UK

**Keywords:** *Aeromonas*, flagella, glycosylation, motility, pseudaminic acid

## Abstract

Bacterial swimming is mediated by the rotation of a flagellar filament. Many bacteria are now known to be able to *O*‐glycosylate their flagellins, the proteins that make up the flagellar filament. For bacteria that use nonulosonic acid sugars such as pseudaminic acid, this glycosylation process is essential for the formation of a functional flagellum. However, the specific role of glycosylation remains elusive. *Aeromonas caviae* is a model for this process as it has a genetically simple glycosylation system. Here, we investigated the localization of the glycans on the *A. caviae* flagellum filament. Using mass spectrometry it was revealed that pseudaminic acid O‐glycosylation was heterogeneous with no serine or threonine sites that were constantly glycosylated. Site‐directed mutagenesis of particular glycosylation sites in most cases resulted in strains that had reduced motility and produced less detectable flagellin on Western blots. For flagellin *O*‐linked glycosylation, there is no known consensus sequence, although hydrophobic amino acids have been suggested to play a role. We, therefore, performed site‐directed mutagenesis of isoleucine or leucine residues flanking the sites of glycosylation and demonstrated a reduction in motility and the amount of flagellin present in the cells, indicating a role for these hydrophobic amino acids in the flagellin glycosylation process.

## Graphical abstract

Flagellin glycosylation is required for swimming motility in *Aeromonas*. Here, we show that O‐glycosylation was heterogeneous with no serine or threonine sites that were constantly glycosylated, and mutation of several sites was found to affect motility. Data herein suggest that hydrophobic amino acids play a role in the glycosylation process.

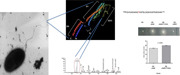

## 1 INTRODUCTION

Within the mesophilic group of aeromonads, several species are significant human pathogens that can cause wound infections and gastroenteritis (Parker & Shaw, 2011). Furthermore, some strains are also the causative agent of the economically important fish disease motile aeromonad septicaemia (MAS) (Hossain et al., 2014). *Aeromonas* express numerous virulence factors to colonize the host including type IV pili (Hadi et al., 2012; Lowry et al., 2014). However, members of the mesophilic aeromonad group are motile in liquid environments by the action of a single polar flagellum, the nanomachine required for locomotion that can also contribute to colonization (Kirov et al., 2004; Lowry et al., 2014). The filament component of the bacterial flagellum is composed of repeating monomer units of subunits called flagellins; for the *Aeromonas* polar flagellum, these are called FlaA and FlaB (Rabaan et al., 2001).

An increasing number of bacteria including *Aeromonas* species have now been shown to be able to *O*‐glycosylate their flagellins, linking sugars onto serine or threonine residues within the central D2/D3 domain of the protein, a region that is thought to form the surface of the flagellar filament (Yonekura et al., 2003). Many of these bacteria use nonulosonic acids such as pseudaminic acid or legionaminic acid or their derivatives (Schirm et al., 2005; Thibault et al., 2001). This glycosylation process is essential for motility and the formation of the flagellar filament and is controlled by the protein Maf (Parker et al., 2012, 2014).

O‐linked flagellin glycosylation has been characterized most extensively in *Campylobacter* species that possess complex glycosylation islands and glycosylate their flagellins with a variety of nonulosonate sugars (Schirm et al., 2005; Thibault et al., 2001). Studies in *Campylobacter jejuni* 81–176 revealed that 19 serine or threonine residues were modified by pseudaminic acid or derivatives, with all but one of these sites being present within the central D2/D3 domain of the protein (Thibault et al., 2001). Furthermore, intact mass spectrometry (MS) analysis of flagellins demonstrated different flagellin components to exist at one time, suggesting heterogeneity (Thibault et al., 2001). However, in *C. jejuni* 81–176 it was established that the identified sites are “usually” occupied, suggesting a small amount of heterogeneity with regard to the site of modification (Thibault et al., 2001).

*Aeromonas caviae* Sch3 attaches the basic form pseudaminic acid (Pse5Ac7Ac) to the central D2/D3 domain of the FlaA and FlaB polar flagellin proteins (Gray et al., 2019; Tabei et al., 2009). The exact sites of flagellin glycosylation have not been identified in *A. caviae*; however, how this bacterium modifies its flagellins may indicate the function of this sugar at the cell surface. For example, if glycosylation is homologous, by always being present on the same serine or threonine residues, it may have a structural role, as flagellin glycosylation is essential for flagellar assembly in a variety of bacteria (Goon et al., 2003; Schirm et al., 2003; Tabei et al., 2009; Twine et al., 2009; Wu et al., 2011). It is also possible that the presence of sugars may also disguise surface‐exposed loop regions of the flagellins from environmental protease cleavage, as *N*‐glycans at the *C. jejuni* cell surface have been shown to potentially protect proteins from gut proteases (Alemka et al., 2013). It could also be required for favorable interactions with host‐cell surfaces, as flagella are essential for bacterial adherence to host cells and therefore the first stages of colonization (Lowry et al., 2014). However, if flagellin glycosylation is heterologous, the movement of pseudaminic acid around the flagellum may disguise this appendage from the host's immune system during infection or it is also possible that flagellin glycosylation may vary depending on the bacterium's situation. *Campylobacter* flagellar glycosylation varies due to phase variation of the glycosyltransferase proteins (Maf proteins) (Van Alphen et al., 2008), of which it has several and is capable of glycosylating its flagella with different nonulosonate sugars (Karkyshev et al., 2002). Furthermore, work by Howard et al. (2009) demonstrated that altered glycosylation levels in *C. jejuni* affect the overall surface charge of the *Campylobacter* flagellum, and in doing so, alters the behavior of a population (Howard et al., 2009).

In contrast to the variety of sugars decorating the polar flagella of *Campylobacter* species, *Helicobacter pylori* glycosylates its polar flagellum solely with Pse5Ac7Ac, similar to *A. caviae* (Schirm et al., 2003). Little heterogeneity was discovered, with FlaA containing six to seven sugars and FlaB, nine to 10 (Schirm et al., 2003). More recently, glycosylation in the organism, *Shewanella oneidensis*, has been investigated, with the flagellins being modified with a sugar related to pseudaminic acid, with five sites of modification being confirmed on the dominant flagellin, FlaB, and four sites on FlaA (Bubendorfer et al., 2013; Sun et al., 2013). Heterogeneity between the sites of glycosylation occupied was not reported in these studies (Bubendorfer et al., 2013; Sun et al., 2013). In addition to glycosylation, these studies also identified methylation on both *S. oneidensis* flagellins; however, sites of methylation did not appear to be essential for either flagellin glycosylation or the production of a functional filament (Bubendorfer et al., 2013; Sun et al., 2013).

Although the biological role of flagellin glycosylation is not well understood, it is clear that bacteria modify their flagella in different ways, and therefore the role of glycosylation may vary in each case. The previous findings that *A. caviae* Sch3 glycosylates its flagellins with six to eight pseudaminic acid residues suggest there to be some heterogeneity in the glycosylation process (Tabei et al., 2009). Here, we further investigated the posttranslational modification of the *A. caviae* polar flagellins, examining the frequency and location of glycosylation within the protein.

## 2 MATERIALS AND METHODS

### 2.1 Bacterial strains, plasmids, and growth conditions

Bacterial strains and plasmids used in this study are listed in Table 1. *Escherichia coli* strains were grown in Luria–Bertani (LB) Miller broth and on LB Miller agar, while *Aeromonas* strains were grown in brain heart infusion broth (BHIB) or on Columbia blood agar (Oxoid). The growth of *Escherichia coli* and *Aeromonas* strains was typically carried out at 37°C. Ampicillin (50 µg/ml), nalidixic acid (50 µg/ml), kanamycin (50 µg/ml), gentamycin (25 µg/ml), streptomycin (50 µg/ml), and chloramphenicol (25 µg/ml) were added when necessary (Table 2).

**Table 1:** Strains and plasmids used in this study

Strain or plasmid	Genotype and use or description	Source or reference
*Escherichia coli* strains		
DH5α	F^−^ Phi80*dlacZ* ΔM15 Δ(*lacZYA*‐*argF*)U169 *deoR recA1 endA1*	Invitrogen
	*hsdR17*(rK‐mK+) *phoA supE*44 lambda‐ *thi*‐1; used for general cloning	
S17‐1λ*pir*	*hsdR pro recA*, RP4‐2 in chromosome, Km::Tn*7* (Tc::Mu) λ*pir*, Tp^r^ Sm^r^	De Lorenzo et al. (1990)
*CC118* λ*pir*	Δ(*ara leu*)*7697 araD139* Δ*lacX74 galE galK phoA20 thi‐1 rspE rpoB*(Rf^r^)	Herrero et al. (1990)
	*argE*(Am) *recA1* λ*pir* ^+^	
*Aeromonas* strains		
*Aeromonas caviae* Sch3N	Sch3, spontaneous Nal^r^	Gryllos et al. (2001)
Plasmids		
pGEMT‐EASY	Cloning vector, Amp^r^	Promega
pBBR1MCS‐5	Broad host range expression vector containing *lac* promoter and*lacI* ^q^, *lacZ*α^+^, and Gm^r^	Kovach et al. (1995)
pBBR1MCS‐5_*flaB*	pBBR1MCS‐5 derivative containing Sch3N *flaB* under the control of its native promoter	This study
pBBR1MCS‐5_*flaB*(T155A)	pBBR1MCS‐5 derivative containing Sch3N *flaB* with a Theronine155 to Alanine point mutation under the control of its native promoter	This study
pBBR1MCS‐5_*flaB*(S159/161A)	pBBR1MCS‐5 derivative containing Sch3N *flaB* with two‐point mutations (Serine159/161Alanine) under the control of its native promoter	This study
pBBR1MCS‐5_*flaB*(S167/169A)	pBBR1MCS‐5 derivative containing Sch3N *flaB* with two‐point mutations (Serine167/169Alanine) under the control of its native promoter	This study
pBBR1MCS‐5_*flaB*(S159/161/167/169A)	pBBR1MCS‐5 derivative containing Sch3N *flaB* with four‐point mutations (Serine159/161/167/169Alanine) under the control of its native promoter	This study
pBBR1MCS‐5_*flaB*(L160A)	pBBR1MCS‐5 derivative containing Sch3N *flaB* with a Leucine160 to Alanine point mutation under the control of its native promoter	This study
pBBR1MCS‐5_*flaB*(I168A)	pBBR1MCS‐5 derivative containing Sch3N *flaB* with an Isoleucine168 to Alanine point mutation under the control of its native promoter	This study

**Table 2:** Primers used in this study

RCL_55	5′‐GCGC**AAGCTT**AGGCAGACTCGTTCAAGCTGT‐3′ (forward)
	Insertion of *Aeromonas caviae* Sch3N *flaB* into pBBR1MCS‐5 contains a ***Hin*dIII** restriction site
RCL_56	5′‐GCGC**GGATCC**CTGCTCCTCATATCAATGATGTTG‐3′ (reverse)
	Insertion of *Aeromonas caviae* Sch3N *flaB* into pBBR1MCS‐5 contains a ***Bam*HI** restriction site
B T155A (R)	5′‐CTAAACCCAAT**GGC**CTGATTGGC‐3′ (reverse)
	To be used in conjunction with RCL_55 to form the front section of *flaB* for overlap extension PCR. Incorporates a T155A site‐directed mutation into FlaB.
B T155A (F)	5′‐GCCAATCAG**GCC**ATTGGGTTTAG‐3′ (forward)
	To be used in conjunction with RCL_56 to form the back section of *flaB* for overlap extension PCR. Incorporates a T155A site‐directed mutation into FlaB.
B S159/161A (R)	5′‐GGCTT**GAG**CCA**AGG**CAAACCCAA‐3′ (reverse)
	To be used in conjunction with RCL_55 to form the front section of *flaB* for overlap extension PCR. Incorporates S159/161A site‐directed mutations into FlaB.
B S159/161A(F)	5′‐TTGGGTTTG**CCT**TGG**CTC**AAGCC‐3′ (forward)
	To be used in conjunction with RCL_56 to form the back section of Sch3N flab for overlap extension PCR. Incorporates S159/161A site‐directed mutations into FlaB.
B S167/169A(R)	5′‐CAATCCC**AGC**AAT**GGC**GAACCCTC‐3′ (reverse)
	To be used in conjunction with RCL_55 to form the front section of *flaB* for overlap extension PCR. Incorporates S167/169A site‐directed mutations into FlaB.
B S167/169A (F)	5′‐GAGGGTTC**GCC**ATT**GCT**GGGATTG‐3′ (forward)
	To be used in conjunction with RCL_56 to form the back section of *flaB* for overlap extension PCR. Incorporates S167/169A site‐directed mutations into FlaB.
B I168A (R)	5′‐CAATCCCAGA**AGC**GCTGAACCCTC‐3′ (reverse)
	To be used in conjunction with RCL_55 to form the front section of *flaB* for overlap extension PCR. Incorporate an I168A site‐directed mutation into FlaB.
B I168A (F)	5′‐GAGGGTTCAGC**GCT**TCTGGGATTG‐3′ (forward)
	To be used in conjunction with RCL_56 to form the back section of *flaB* for overlap extension PCR. Incorporates an I168A site‐directed mutation into FlaB.
B L160A(R)	5′‐GGCTTGAGA**CGC**GCTAAACCCAA‐3′ (reverse)
	To be used in conjunction with RCL_55 to form the front section of *flaB* for overlap extension PCR. Incorporates an L160A site‐directed mutation into FlaB.
B L160A (F)	5′‐TTGGGTTTAGC**GCG**TCTCAAGCC‐3′ (forward)
	To be used in conjunction with RCL_56 to form the back section of *flaB* for overlap extension PCR. Incorporates an L160A site‐directed mutation into FlaB.

*Note*: Restriction sites or changed codons for site directed mutagenesis are indicated in bold type.

### 2.2 General DNA methods

Where required DNA restriction endonucleases, T4 DNA ligase, and alkaline phosphatase were used as recommended by the suppliers (NEB).

### 2.3 Generation of pBBR1MCS‐5_flaB and site‐directed mutants

The *flaB* gene (918 bp), encoding the *A. caviae* Sch3 polar flagellin (FlaB) (Gray et al., 2019), was cloned into pBBR1MCS‐5 along with a region upstream of the transcription start site to allow expression of *flaB* from its native promoter (99 bp). Q5 high‐fidelity DNA polymerase (NEB) was used to amplify *flaB* from Sch3 *A. caviae* genomic DNA with the primers RCL_55 and RCL_56. The PCR product was directly cut with a combination of *Hin*dIII and *Bam*HI restriction enzymes before being ligated into *Hin*dIII/*Bam*HI cut pBBR1MCS‐5 (Kovach et al., 1995). The ligation was transformed into chemically competent *E. coli* DH5α and minipreps were carried out with resulting transformants to isolate the vector DNA. The presence of *flaB* was investigated with a *Bam*HI restriction digest and agarose gel electrophoresis (compare with linearized empty pBBR1MCS‐5). Likely pBBR1MCS‐5_*flaB* constructs were sent for sequencing using M13 forward and reverse primers to confirm the presence of *flaB*. Site‐directed mutants of FlaB glycosylation sites were generated to determine whether particular glycosylation sites were important for the motility of *A. caviae*; FlaB site‐directed mutants were created via overlap extension PCR (OE‐PCR), where serine and threonine residues on the peptide, _146_FQVGADANQTIGFSLSQAGGFSISGIAK_173_, were mutated to alanine residues, due to the structural simplicity and nonpolar properties of this amino acid.

### 2.4 Motility assays

To assess the motility of *Aeromonas* strains, bacterial colonies were transferred with a sterile toothpick into the center of motility agar plates (1% tryptone, 0.5% NaCl, 0.25% agar). The plates were incubated face up at 25°C for 14–24 h, and motility was assessed by examining the migration of bacteria through the agar from the center toward the periphery of the plate.

### 2.5 Flagellin purification method

To purify *A. caviae* polar flagellins, a flagellar shearing method, adapted from Wilhelms et al. (2012), was carried out as described by Lowry et al. (2015). Briefly, *Aeromonas* strains were grown on large swarm agar plates and flagella were sheared from the cells via the use of a blender for 10 min. Cells were pelleted by centrifugation at 8000*g* for 30 min and debris removed from the supernatant by further centrifugation at 18,000*g* for 20 min. Flagella were pelleted via centrifugation at 75,000*g* for 1.5 h and resuspended in phosphate‐buffered saline.

### 2.6 Sodium dodecyl sulfate‐polyacrylamide gel electrophoresis (SDS‐PAGE) and immunoblotting

SDS‐PAGE and immunoblotting of *Aeromonas* whole‐cell preparations were carried out as previously described (Tabei et al., 2009). *Aeromonas* strains were grown overnight in BHIB at 37°C. Equivalent numbers of cells were harvested by centrifugation. Protein precipitation of supernatant proteins was carried out by adding two volumes of ice‐cold ethanol to the desired proteins (in solution) and incubating on ice for 1–3 h. Precipitated proteins were pelleted at 3900*g* for 30 min at 4ᵒC. After centrifugation, the supernatant was discarded and the protein pellet was allowed to dry in the air for 10 min. Cell pellets or pelleted supernatants were boiled in SDS‐PAGE loading buffer for 5 min. Protein samples were separated on SDS‐polyacrylamide gels (12% acrylamide). For immunoblotting, proteins were transferred onto a Hybond‐C (GE Healthcare) nitrocellulose membrane. Following the transfer, membranes were blocked with 5% (w/v) powdered skimmed milk. For identification of flagellin, membranes were probed with a polyclonal rabbit anti‐polar flagellin antibody (1:10,000) that only recognizes glycosylated flagellin or a rat anti‐polar flagellin antibody (1:1,000) that recognizes both glycosylated and unglycosylated flagellin (Parker et al., 2014). A goat anti‐rabbit or goat anti‐rat horseradish peroxidase‐conjugated secondary antibody (1:5000) was used in combination with the ECL detection system (GE Healthcare) before being exposed to X‐ray film and developed using a Compact X4 automatic film processor (Xograph Healthcare).

### 2.7 Statistical analysis

The differences between the wild‐type and mutant strains and the mutant strains versus the complemented strains were analyzed using GraphPad Prism 5.0 (GraphPad Software). Data are given as means ± the standard error of the mean (SEM). Statistical significance was compared to the wild‐type by *t* test or one‐way analysis of variance (Tukey's multiple comparisons test).

### 2.8 Trypsin digest of flagellin

In‐gel trypsin digestion was performed as previously described (Couto et al., 2011; Shevchenko et al., 2006). Gel bands of interest were excised from a polyacrylamide gel after SDS‐PAGE and transferred to clean LoBind Eppendorf tubes. Gel pieces were de‐stained with 100 mM ammonium bicarbonate/acetonitrile solution (50:50, v/v) and dehydrated with 100% acetonitrile. Reduction and alkylation were carried out with 10 mM of dl‐dithiothreitol and 55 mM iodoacetamide, respectively. In‐gel digestion with trypsin (Promega) was performed at a ratio of 20:1 (protein:enzyme; w/w) (0.5 μg) in 100 mM ammonium bicarbonate 5% (v/v) formic acid at 37°C for 16 h. After digestion, peptides were extracted and dried in a vacuum centrifuge and stored at −20°C

### 2.9 Liquid chromatography with tandem MS (MS/MS) analysis of flagellins

A U3000 nanoflow high‐performance liquid chromatography (HPLC) system (Thermo Fisher Scientific) was directly connected to the mass spectrometer (maXis^TM^ UHR‐Qq‐ToF; Bruker Daltonic), fitted with an EZ Nanoflow Electrospray needle. The U3000 HPLC system was equipped with a nanoLC analytical column (75 μm × 15 cm packed with C18 material, 5 µm, 100 Å particles; LC Packings) and micro precolumn (300 µm i.d. × 5 packed with C18 material, 5 µm, 100 Å particles; LC Packings) at flow rates of 300 nl/min and 30 μl/min, respectively. A linear HPLC gradient using buffer A (97% [v/v] HPLC water, 3% [v/v] HPLC acetonitrile with 0.1% [v/v] formic acid) and buffer B (97% [v/v] HPLC acetonitrile, 3% [v/v] HPLC grade water with 0.1% [v/v] formic acid), using a 120 min program, was applied as follows: 0% B (0–5 min), 0%–35% B (5–95 min), 35%–100% B (95–101 min), 100% B (101–106 min), 0% B (106–120 min). Acquired data were analyzed initially using EasyProt (Gluck et al., 2013) and then further analyzed manually using the mass of precursor/fragment ions predicted from in silico digests using the protein product tool on the Protein Prospector website (http://prospector.ucsf.edu/prospector/mshome.htm).

## 3 RESULTS

### 3.1 *Aeromonas caviae* flagellins are heterogeneously glycosylated

**Figure 1:**
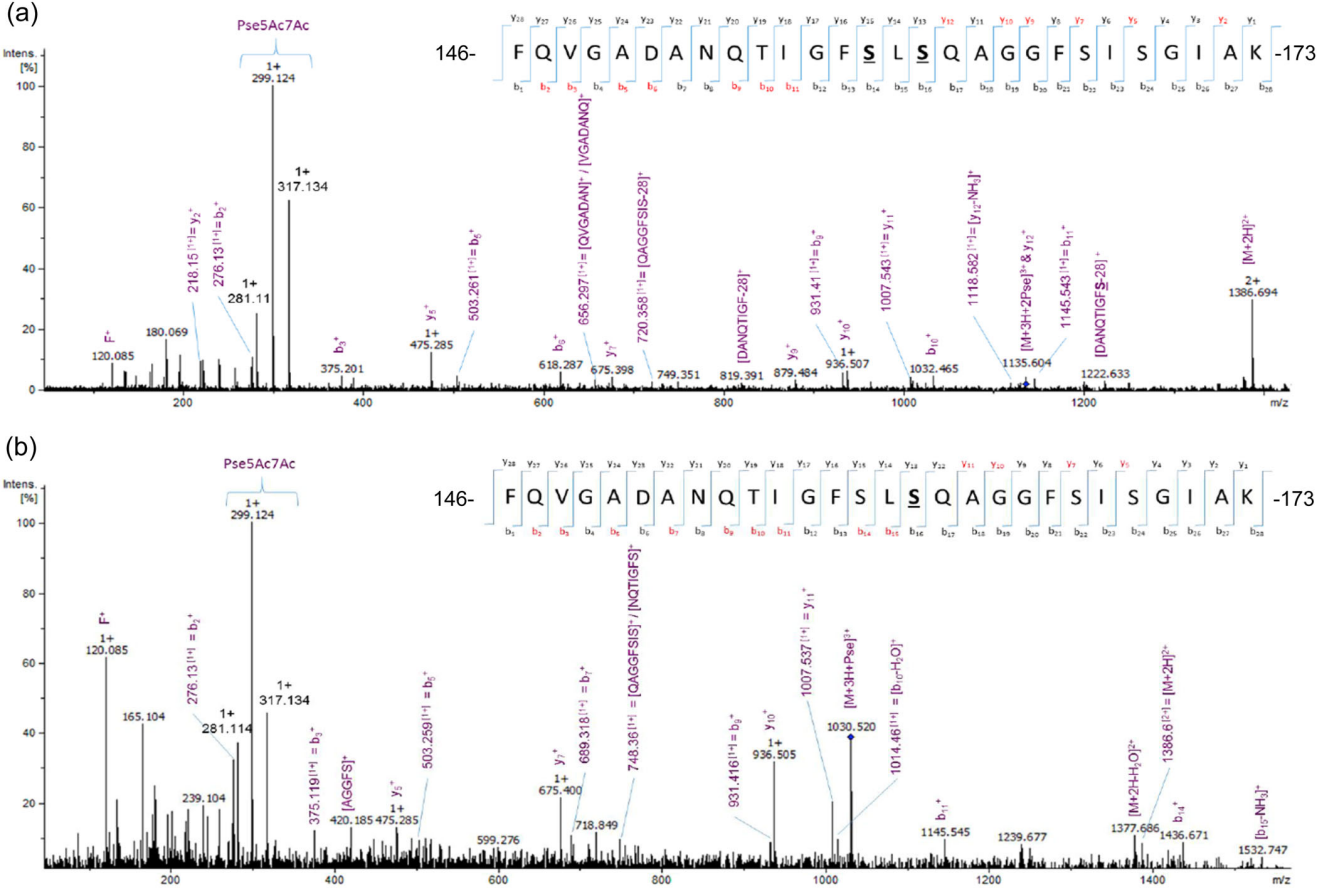
FlaB peptide can be found with either one or two pseudaminic acid residues attached. (a) Collision‐induced dissociation MS/MS (CID‐MS/MS) spectra of the triply charged ion, *m*/*z* 1135.2, which corresponds to the FlaB peptide, _146_FQVGADANQTIGFSLSQAGGFSISGIAK_173_, containing two pseudaminic acid residues at serine 159 and serine 161. **S** denotes the positions of the sugar and the red text refers to the y and b ions present in the spectra. This particular peptide was eluted at 86.24 min. (b) CID‐MS/MS spectra of the triply charged ion, *m*/*z* 1029.8, which corresponds to the FlaB peptide, _146_FQVGADANQTIGFSLSQAGGFSISGIAK_173_, containing one pseudaminic acid residue at serine 161. **S** denotes the positions of the sugar and the red text refers to the y and b ions present in the spectra. This particular peptide was eluted at 80.82 min.

Previous studies have shown the *A. caviae* Sch3 polar flagellins FlaA and FlaB are decorated with between six and eight residues of Pse5Ac7Ac; these are located within the D2/D3 domain of the flagellin, O‐linked onto 19 potential serine or threonine residues (Tabei et al., 2009). Attempts were made to determine if particular residues were constantly glycosylated or whether glycosylation was heterogeneous. Therefore, in‐gel trypsin digestions of *A. caviae* flagellin samples were carried out to yield manageable FlaA/B peptides for MS analysis. As *A. caviae* FlaA and FlaB are 95% identical at the amino acid level, it was decided to concentrate on FlaB only to remove any confusion with peptide identification. This was achieved by expressing *flaB* only in an *A. caviae flaA*/B mutant background. Collision‐induced dissociation MS/MS (CID‐MS/MS) was carried out and mass spectra obtained were subjected to both manual and Easyprot analysis (Gluck et al., 2013). CID‐MS/MS was found to preferentially fragment the pseudaminic acid on glycopeptides present, which could be visualized in the MS/MS spectra as large, singly charged, diagnostic ion peaks at *m*/*z* 317.1 and 299.1 (Figure 1), corresponding to pseudaminic acid and the sugar minus water, respectively, as demonstrated previously (Parker et al., 2014; Thibault et al., 2001). Although many glycopeptides could be manually visualized within the spectra (recorded between 60 and 100 min retention times), many difficulties were faced when trying to identify them. Due to the lack of lysine and arginine residues, tryptic peptides within this D2/D3 region are large; therefore, there are many possible sites of glycosylation on each tryptic peptide. Other enzymes such as LysC, GluC, and chymotrypsin did not cut frequently enough on their own, but when used in combination, yielded peptides too small to be considered by the mass spectrometer. The FlaB peptide (amino acid 146–173) was the most frequent glycopeptide observed when analyzing the spectra manually; this was present at *m*/*z* 1135.6[3+] (Figure 1a) corresponding to this peptide containing two pseudaminic acids and at *m*/*z* 1030.5[3+] (Figure 1b), equivalent to the peptide containing one pseudaminic acid. The exact sites of glycosylation could not be determined without the aid of EasyProt due to the preferential fragmentation of pseudaminic acid via CID‐MS/MS; the peaks corresponding to the rest of the peptide fragmentation were, therefore, extremely small and largely lost in the spectra's background noise. EasyProt confirmed that the FlaB peptide, _146_FQVGADANQTIGFSLSQAGGFSISGIAK_173_, is glycosylated either once or twice, and is never found in its unglycosylated form. EasyProt analysis demonstrated that the exact sites of glycosylation appear to vary on this peptide. When the FlaB peptide was glycosylated once, this was on either threonine 155, serine 159, serine 161, serine 167, or serine 169. When glycosylated twice (which was more common), the modification of both central serine residues (serine 159 and 161) was observed most frequently. However, the peptide was also present with modifications on both threonine 155 and serine 159. Furthermore, there were also many glycopeptides present that only appeared once in the spectra and could not be assigned to a single peptide. This may demonstrate that *A. caviae* flagellin glycosylation is extremely variable, with many different glycopeptides, or versions of the same peptide, being present in a wild‐type flagellin sample.

### 3.2 The *Aeromonas caviae* flagellin is also methylated

**Figure 2:**
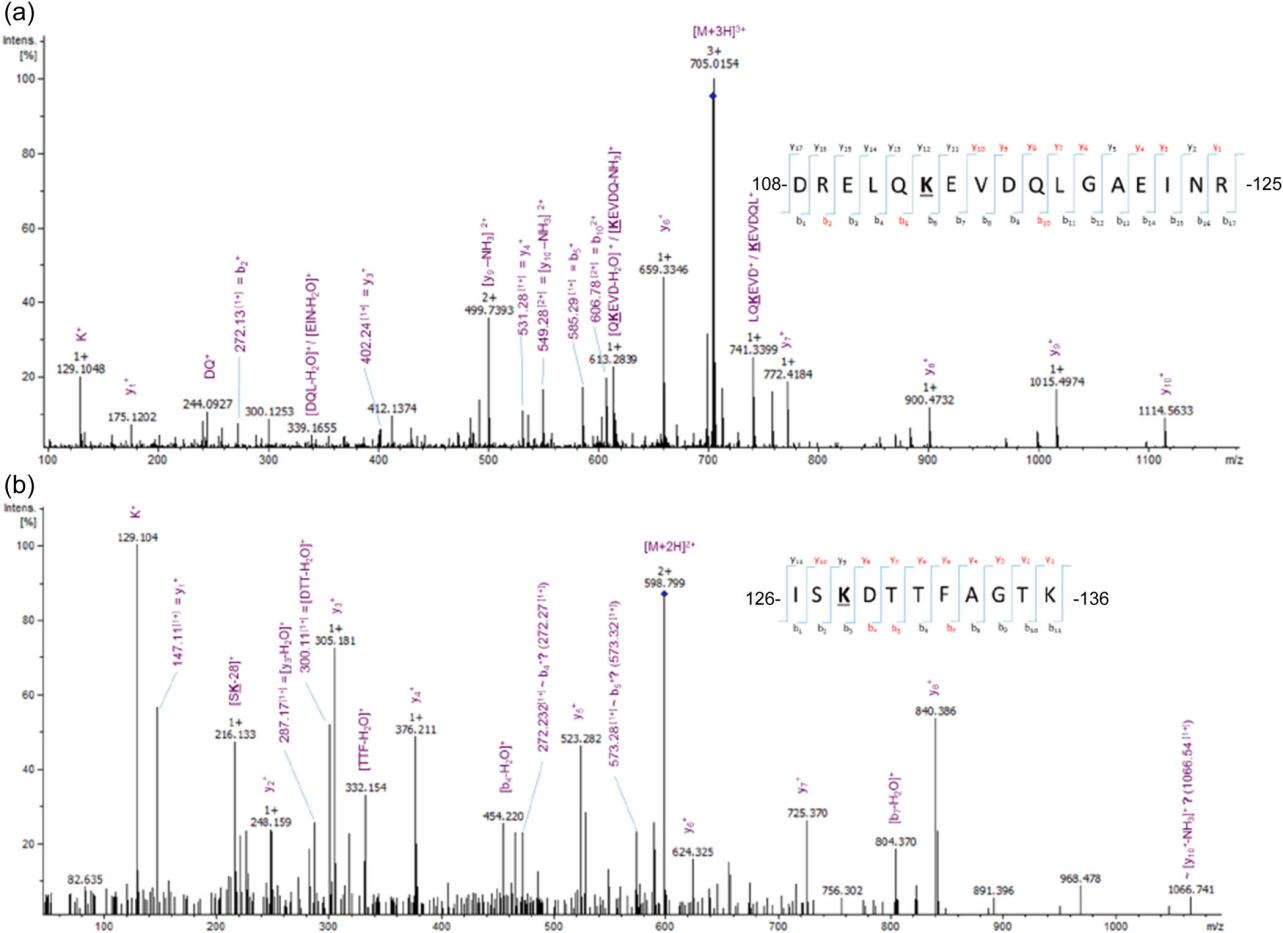
Peptides of the flagellin FlaB are methylated. (a) Collision‐induced dissociation MS/MS (CID‐MS/MS) spectra of the triply charged ion, *m*/*z* 704.69, which corresponds to the dimethylated FlaA peptide, 108DREALQKEVDQLGAEINR125. **K** denotes the modified residue and the red text refers to the y and b ions present in the spectra. This particular peptide was eluted at 44.29 min. (b) CID‐MS/MS spectra of the doubly charged ion, *m*/*z* 598.8, which corresponds to the dimethylated FlaB peptide, 126ISKDTTFAGTK136. **K** denotes the modified residue and the red text refers to the y and b ions present in the spectra. This particular peptide was eluted at 28.87 min.

In other bacteria such as *Shewanella*, lysine residues of flagella have been demonstrated to be methylated (Sun et al., 2013). Therefore, methylation of lysine residues was also investigated in *A. caviae*. When methylation of lysine residues (methyl/dimethyl/trimethyl) was introduced into the EasyProt search parameters, a variety of methylated peptides were discovered. The FlaA peptide, _108_DREALQKEVDQLGAEINR_125_, was suggested to be present in numerous forms in the wild‐type *A. caviae* flagellin sample, as evidence for dimethylation and trimethylation on the central lysine 114 was discovered, along with the unmodified peptide (Figure 2a). Furthermore, EasyProt analysis of flagellin also suggested the FlaB peptide, _126_ISKDTTFAGTK_136_ (Figure 2b), to be dimethylated on lysine 128 and the peptide, _38_INSAKDDAAGLQISNR_53_ (data not shown), present in both FlaA and FlaB, to be di‐ and trimethylated on lysine 42. Both peptides were discovered in their unmodified forms also.

### 3.3 Removal of glycosylation sites affects motility

As the FlaB peptide, _146_FQVGADANQTIGFSLSQAGGFSISGIAK_173_, was identified to be glycosylated (potentially with either one or two pseudaminic acid residues), and the site of glycosylation appears to vary, FlaB was selected for site‐directed mutagenesis studies to investigate whether any specific glycoforms of this peptide are particularly required for the motility of *A. caviae*. These studies were carried out in an *A. caviae flaA flaB* double mutant background strain (Rabaan et al., 2001) into which the wild‐type copy or site‐directed mutants were introduced. A single mutation was made in T155, double mutations in S159/161 or S167/169, and quadruple mutations in S159/161/167/169. The wild‐type copy and site‐directed *flaB* mutants were cloned separately into the broad host range vector pBBR1MCS‐5.

The plasmid, pBBR1MCS‐5*_flaB*, and the vectors containing desired site‐directed mutations (T155A, S159/161A, S167/169A, or S159/161/167/169A) were conjugated into an *A. caviae flaAB* mutant (Rabaan et al., 2001). Swimming motility assays were carried out on large swimming motility plates, allowing an *A. caviae flaAB* mutant expressing the desired FlaB site‐directed mutant to be analyzed alongside the *flaAB* mutant alone, the mutant containing the empty vector, and the mutant containing wild‐type *flaB*.

**Figure 3:**
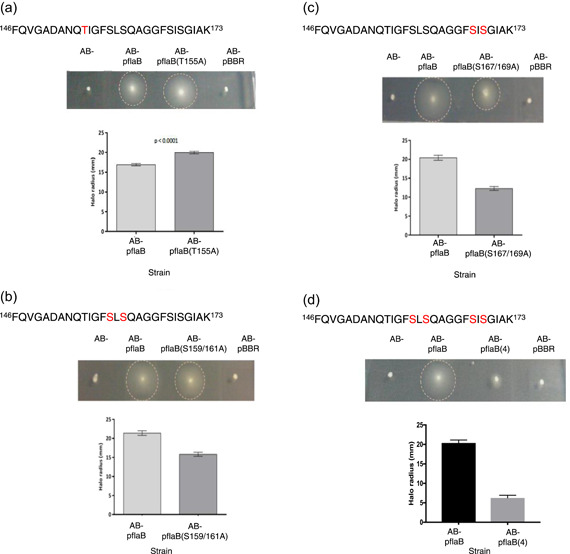
Site‐directed mutants of particular serine or threonine residues affect motility. Analysis of a FlaB site‐directed mutants in an *A. caviae flaAB* mutant background. (a) FlaB T155A. (b) FlaB S159/161A site‐directed double mutant*.* (c) FlaB S167/169A site‐directed double mutant. (d) FlaB S159/161/167/169A site‐directed quadruple mutant. For each experiment swimming motility assays were carried out on 0.25% (w/v) agar for an *A. caviae flaAB* mutant (*AB−*), a *flaAB* mutant containing pBBR1MCS‐5_*flaB* (*AB−* p*flaB*), a *flaAB* mutant containing pBBR1MCS‐5 and the site‐directed allele, for example, [*AB−* p*flaB*(T155A)] and a *flaAB* mutant containing empty pBBR1MCS‐5 (*AB−* pBBR). The radius of each motility halo was measured after 24 h and average measurements are presented here (*n* = 6) plus or minus the standard error of the mean. *p* < 0.0001 was generated when a paired *t* test was carried out on the data sets.

**Figure 4:**
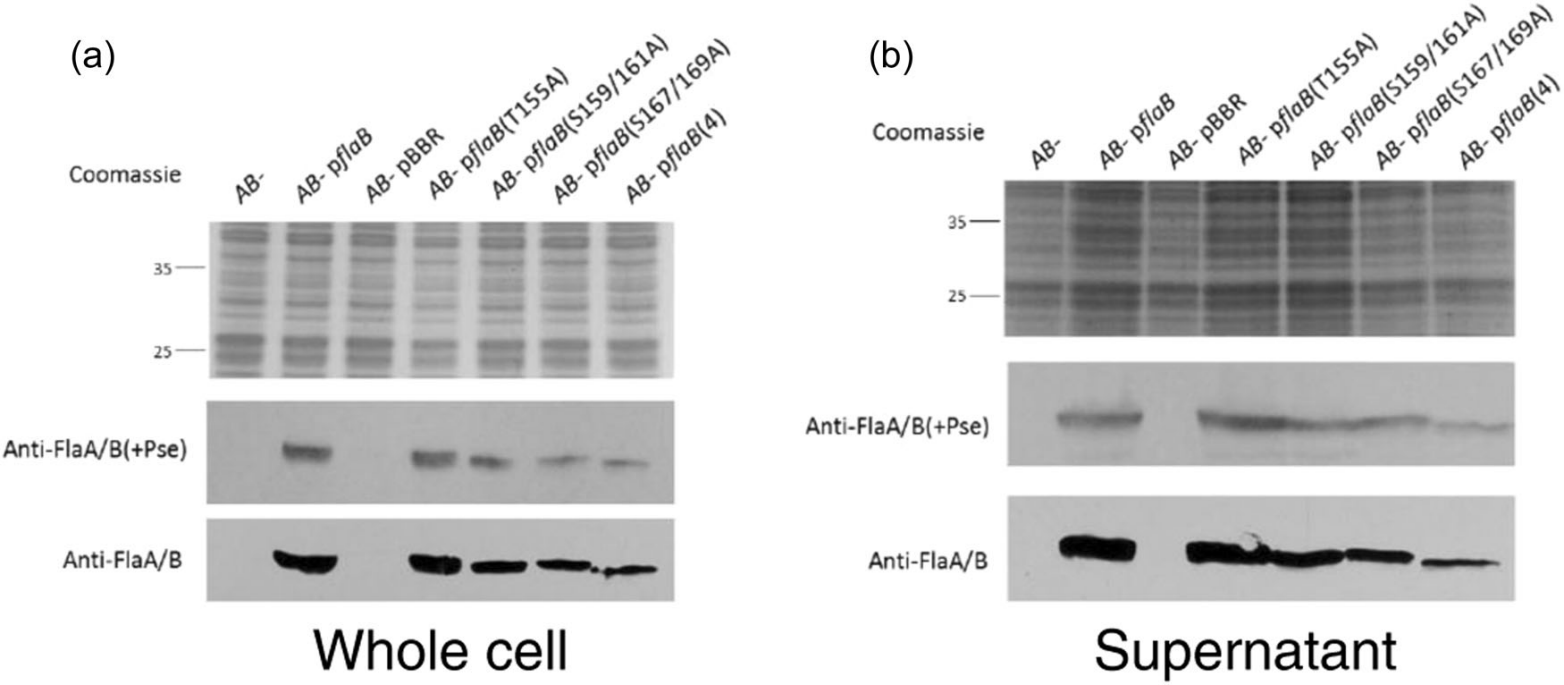
Site‐directed mutants of particular serine or threonine residues affect flagellin production. Western blot analysis of *Aeromonas caviae* whole‐cell samples (a) and supernatant samples (b). Both samples from (a and b) were probed with a rabbit, antipolar flagellin antibody that recognizes only glycosylated flagellin [anti‐FlaA/B(+Pse)], and a rat anti‐polar flagellin antibody that recognizes both glycosylated and unglycosylated forms of flagellin (anti‐FlaA/B). In each case: lane 1, the *A. caviae flaAB* mutant (*AB−*); lane 2, the *flaAB* mutant containing pBBR1MCS‐5_*flaB* (*AB−* p*flaB*); lane 3, the *flaAB* mutant containing empty pBBR1MCS‐5 (*AB−* pBBR); lane 4, the *flaAB* mutant containing pBBR1MCS‐5_*flaB*(T155A) [*AB−* p*flaB*(T155A)]; lane 5, the *flaAB* mutant containing pBBR1MCS‐5_*flaB*(S159/161A) [*AB−* p*flaB*(S159/161A)]; lane 6, the *flaAB* mutant containing pBBR1MCS‐5_*flaB*(S167/169A) [*AB−* p*flaB*(S167/169A)]; lane 7, the *flaAB* mutant containing pBBR1MCS‐5_*flaB*(S159/161/167/169A) [*AB−* p*flaB*(4)].

As expected, the *A. caviae flaAB* mutant alone was nonmotile as was the version complemented with the vector pBBR1MCS‐5 only. When the wild‐type version of the FlaB was introduced into the mutant motility was restored. The *flaB*(T155A) construct was able to complement the nonmotile phenotype of the *A. caviae flaAB* mutant and was found to swim significantly more (18% more) than the mutant expressing wild‐type FlaB (Figure 3a). Expression of the pBBR1MCS‐5 constructs containing the double mutations, *flaB*(S159/161A) and *flaB*(S167/169A), were both found to restore motility in the *flaAB* mutant; however, the motility was significantly impaired (Figure 3b). These strains displayed a reduction in motility of 26% (S159/161) and 39% (167/169) compared to the motility levels of the *flaAB* mutant containing the wild‐type FlaB (Figure 3b,c). When the motility of the quadruple mutant construct, *flaB*(S159/161/167/169A), was analyzed, severely impaired motility was visualized after 24 h; this strain was therefore classed as nonmotile (Figure 3d). Western blot analysis was carried out on whole‐cell (Figure 4a) and precipitated supernatant (Figure 4b) site‐directed mutant flagellin samples, to compare the mutant flagellins to wild‐type FlaB. Each sample was probed with an antibody that recognizes glycosylated flagellin only [anti‐FlaA/B(+Pse)], and an antibody that recognizes both glycosylated and unglycosylated flagellin forms (anti‐FlaA/B) (Figure 4). When wild‐type FlaB was analyzed, a thick band resulted, most likely due to the flagellins being present in different glycoforms, with some possessing only six pseudaminic acid residues and others seven or eight sugars (Figure 4) (Tabei et al., 2009). When the site‐directed mutant flagellins were analyzed via Western blot analysis, FlaB(T155A) displayed a band thickness comparable to the wild‐type flagellin when normalized whole‐cell samples and the supernatant samples were analyzed with both anti‐FlaA/B and anti‐FlaA/B(+Pse). Therefore, it is likely that FlaB(T155A) was glycosylated to levels similar to the wild‐type flagellin. However, although both FlaB(S159/161A) and FlaB(S167/169A) can produce a functional flagellum, they are observed as thinner bands on the Western blots compared to wild‐type FlaB; this is potentially due to only possessing the lower levels of flagellin glycosylation (i.e., restricted to possessing only six to seven pseudaminic acid residues, whereas wild‐type flagellin is free to occupy more sites). Additionally, the levels of both FlaB(S159/161A) and FlaB(S167/169A) visually appear to be lower than the levels of wild‐type FlaB or FlaB(T155A). Furthermore, a noticeable size shift can be observed when wild‐type FlaB samples, FlaB(T155A), FlaB(S159/161A), and FlaB(167/169A), are compared to the quadruple FlaB mutant flagellin sample (Figure 4). This is apparent both in the whole‐cell and supernatant Western blots (Figure 4) and demonstrates that although the quadruple mutant flagellin is still glycosylated and exported, it is either not incorporated into a functional flagellar filament or any filament formed has an impaired function.

The mutated FlaB(S159/161A) flagellin was selected for analysis via CID‐MS/MS, due to these serine residues being the most regularly occupied sites on the wild‐type flagellin (observed by EasyProt). EasyProt analysis of the data suggested that when serine 159 and serine 161 are comutated to alanine residues, the new peptide, _146_FQVGADANQTIGFALAQAGGFSISGIAK_173_, is still glycosylated, but only with one sugar. EasyProt detected the triply charged ion, *m*/*z* 1019.18, corresponding to this peptide modified with one sugar. This sugar can occupy any of the remaining serine or threonine sites (T155, S167, or S169), with a pseudaminic acid at serine 167 being the predominant glycoform.

### 3.4 Mutation of D2/D3 domain hydrophobic amino acids affects motility

**Figure 5:**
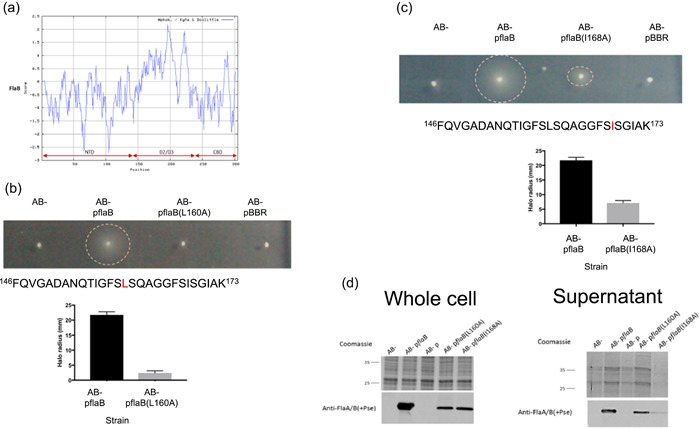
The presence and mutation of hydrophobic amino acids in the *Aeromonas caviae* Sch3 polar flagellin, FlaB. (a) A Kyte and Doolittle hydrophobicity plot displaying the hydrophobicity of the FlaB N‐terminal domain (NTD), D2/D3 domain, and the chaperone binding domain (CBD) (scored by the Kyte–Doolittle scale). (b) Analysis of a FlaB L160A site‐directed mutant in an *A. caviae flaAB* mutant*.* (c) Analysis of a FlaB I168A site‐directed mutant in an *A. caviae flaAB* mutant. For each experiment (b and c), swimming motility assays were carried out on 0.25% (w/v) agar for an *A. caviae flaAB* mutant (*AB−*), a *flaAB* mutant containing pBBR1MCS‐5_*flaB* (*AB‐* p*flaB*), a *flaAB* mutant containing pBBR1MCS‐5 and the site‐directed allele, for example, [*AB−* p*flaB*(I1681A)], and a *flaAB* mutant containing empty pBBR1MCS‐5 (*AB−* pBBR). The radius of each motility halo was measured after 24 h and average measurements are presented here (*n* = 6) plus or minus the standard error of the mean. *p*< 0.0001 was generated when a paired *t* test was carried out on the data sets. (d) Western blot analysis of *Aeromonas caviae* whole‐cell samples and supernatant samples. Both samples were probed with a rabbit antipolar flagellin antibody that recognizes only glycosylated flagellin [anti‐FlaA/B(+Pse)] and a rat antipolar flagellin antibody that recognizes both glycosylated and unglycosylated forms of flagellin (anti‐FlaA/B). In each case: lane 1, the *A. caviae flaAB* mutant (*AB−*); lane 2, the *flaAB* mutant containing pBBR1MCS‐5_*flaB* (*AB−* p*flaB*); lane 3, the *flaAB* mutant containing empty pBBR1MCS‐5 (*AB−* pBBR); lane 4, the *flaAB* mutant containing pBBR1MCS‐5_*flaB*(L160A) [*AB−* p*flaB*(L160A)]; lane 5, the *flaAB* mutant containing pBBR1MCS‐5_*flaB*(I168A) [*AB−* p*flaB*(I168A)]; lane 6, the *flaAB* mutant containing pBBR1MCS‐5_*flaB*(S208/210A) [*AB−* p*flaB*(S208/210A)].

For N‐linked glycosylation, there is a known consensus sequence and there is no known consensus sequence for O‐linked flagellin glycosylation (Nothaft & Szymanski, 2010). Several studies have recognized that the specific sites of glycosylation appear to occur in highly hydrophobic regions of the protein, suggesting there to be some selectivity to the glycosylation process (Schirm et al., 2003; Thibault et al., 2001). The Kyte and Doolittle hydrophobicity plot in Figure 5a shows that the hydrophobic amino acids in flagellin FlaB are clustered in the D2/D3 domain. Furthermore, these regions of hydrophobicity are most commonly separated by serine or threonine residues, unlike the rest of the protein. The sites of glycosylation identified here, on the FlaB tryptic peptide [146–173], are surrounded by hydrophobicity, with the commonly comodified serine 159 and serine 161, being separated by a leucine residue. Furthermore, these serine residues are also flanked by hydrophobic residues. Although serine 167 and 169 are not found comodified, they are individually targeted for glycosylation (from EasyProt analysis) and are separated by an isoleucine residue, as well as being flanked by hydrophobic regions (although not quite as hydrophobic as serine 159/161). The less commonly modified threonine 155 precedes a hydrophobic amino acid sequence; however, it is itself, preceded by acidic and polar amino acids. This may indicate why the mutation of this residue is not detrimental to *A. caviae* motility and why the modification of this residue is not as common as the central serine residues. The hydrophobic amino acids, L160 and I168, separating the serine residues on the FlaB peptide [146–173], were targeted for site‐directed mutational studies. The residues were individually mutated to the less hydrophobic and similarly sized amino acid, alanine.

FlaB site‐directed mutants were created by overlap‐extension PCR and cloned into pBBR1MCS‐5. Both the leucine 160 and isoleucine 168 point mutations individually had a detrimental effect on *A. caviae* motility. The mutant FlaB containing the leucine mutation (L160A), could not restore the motility of a *flaAB* mutant after 24 h (Figure 5b). When the isoleucine FlaB mutant (I168A) was introduced into the *A. caviae flaAB* mutant, only minimal amounts of motility were present, showing the I168A mutation to have a serious, detrimental effect on the motility of *A. caviae* (Figure 5c).

The FlaB site‐directed mutant flagellins were also subjected to Western blot analysis. All mutated flagellins were shown to be present in their glycosylated form [when probed with anti‐FlaA/B(+Pse)], but only very low levels of FlaB(L160A) and FlaB(I168A) were detected, and these levels could not be detected in the whole‐cell samples using anti‐FlaA/B that recognizes both forms of the flagellins (Figure 5d). Furthermore, even though FlaB(L160A) and FlaB(I168A) caused impaired motility in *A. caviae*, glycosylated flagellins were still found to be exported but only in small amounts (Figure 5d). Although these samples could not be normalized due to the precipitation of proteins, it is likely that these proteins are present in smaller amounts due to the levels observed in whole‐cell samples (Figure 5d). Additionally, the detection of these mutated flagellins in supernatant samples with the anti‐FlaA/B antibody shows that these mutations do not affect antibody recognition of these proteins and that extremely low protein levels are why this antibody could not detect these mutated proteins in whole‐cell samples (Figure 5d).

The flagella shearing method was carried out in an attempt to purify glycosylated FlaB(L160A) and FlaB(I168A) for MS analysis. Only very small amounts of flagella could be pelleted in each case by this method. Samples were tryptically digested and run on the maXisTM Q‐TOF for CID‐MS/MS analysis of the samples; however, no glycopeptides could be detected via manual analysis and EasyProt detected limited peptides for each mutant. Therefore, analysis of the glycosylation of these flagellins could not be carried out due to being unable to obtain a sufficient amount of protein. This, however, could indicate that although these mutated flagellins are still glycosylated and exported, these mutations may have a severe effect on flagellar assembly, or export, and may be the reason for the lack of flagella pelleted during the shearing method of flagellin purification.

## 4 DISCUSSION

To analyze the glycosylation states of the *A. caviae* Sch3 flagellin, CID‐MS/MS was chosen as this allowed both manual and EasyProt analysis of the data (Gluck et al., 2013). Trypsin digestion of the D2/D3 domain (the glycosylated region of interest) resulted in large, heavy, tryptic peptides, making analysis of this region problematic. However, analysis of the FlaB [144–173] tryptic peptide provided interesting findings demonstrating the heterogeneous nature of O‐glycosylation and that no serine or threonine sites are constantly glycosylated.

Manual analysis of wild‐type *A. caviae* flagellins by CID‐MS/MS demonstrated that many glycopeptides are present in a sample. Some eluted multiple times, whereas others were only detected once, which may show the heterogeneity of *A. caviae* flagellin glycosylation. However, one glycopeptide was confidently identified from FlaB: _146_FQVGADANQTIGFSLSQAGGFSISGIAK_173_. It was observed that this peptide can be glycosylated with either one or two pseudaminic acid residues and that the site of modification can vary on the peptide. Glycosylation of the two central serine residues (serine 159 and 161) was detected most frequently. This peptide was never found in its unmodified form in wild‐type flagellin samples, suggesting that the glycosylation of this D2/D3 domain peptide, in particular, is important for the formation of a functional flagellar filament. This was supported by the site‐directed mutagenesis studies, where the double mutations at S159/161A and S167/169A, caused a significant decrease in *A. caviae* motility and less flagellin protein being present. This suggests that these mutant flagellins may fold in a way that makes them more readily degraded, maybe due to their less efficient polymerization, or they are less stable compared to the wild‐type flagellins. Mass spectrometric analysis of the mutated flagellin, FlaB(S159/161A), uncovered that the mutated FlaB [146–173, S159/161A] peptide was still glycosylated, but only ever with a single sugar. Here, pseudaminic acid occupied any of the remaining serine and threonine sites, with serine 167 being identified as the most frequent. It is, therefore, possible that the decreased *A. caviae* motility observed when only this mutated flagellin is present, maybe due to the flagella formed having a decreased hydrophilicity, and therefore impaired ability to move through an aqueous environment. In addition, it has previously been noted that unglycosylated flagellin is recognized less by the chaperone, FlaJ (Parker et al., 2014). Therefore, reduced flagellin glycosylation may lead to a reduced recognition of the flagellins by FlaJ, leading to decreased flagellin export and therefore filament polymerization. However, there is also less protein present within the bacterial cell, which may affect flagellar assembly and filament length, suggesting that there may be protein degradation in the less glycosylated versions. Site‐directed mutagenesis studies of the 19 sites of glycosylation in *C. jejuni* have also shown specific glycosylation sites to have an impact on motility, where the individual mutation of three serine residues in particular (in *C. jejuni* 81–176) caused the formation of truncated flagellar filaments and therefore reducing bacterial motility (Ewing et al., 2009). Although single site‐specific mutants of serine had little effect on motility, this was only seen when one or more residues were mutated. Conversely, mutation of the single threonine (T155A) caused an increase in motility, this could be due to optimal folding or increased flagellin stability. Impaired motility is also observed when the mutated flagellin, FlaB(S167/169A), is the only flagellin present. The two mutated residues here were never found to be comodified in wild‐type flagellin samples, and therefore it is likely that the serine residues, 159 and 161, are still the predominantly modified residues on the FlaB peptide [146–173, S167/169A]. As impaired motility is also seen with the comutation of these residues, it is possible that this phenomenon is not related to flagellin glycosylation, but occurs due to the increased hydrophobicity of the peptide (from mutating serine to alanine and the loss of Pse5Ac7Ac), resulting in an overall increase in the hydrophobicity of the polar flagellum. This may lead to less favorable interactions with the aqueous environment.

When all four serine residues on this peptide (S159, S161, S167, and S169) were mutated, the flagellin was found to be smaller than the wild‐type and other site‐directed mutant flagellins. Furthermore, the size of this flagellin also demonstrates that when this region of the protein is not glycosylated it cannot be compensated for by glycosylation of the other remaining sites. This further indicates that this region is essential for motility. The mutation of these residues may result in the improper folding of the flagellin, or these flagellins may polymerize differently from the wild‐type flagellins and be the reason for the impaired and nonmotile phenotypes visualized here. Although the D2/D3 region in other bacterial flagellins allows large deletions or additions within this domain with little effect on filament formation (Reid et al., 1999; Szabo et al., 2011).

Previous studies into bacterial O‐linked flagellin glycosylation have suggested that local regions of hydrophobicity may play a role in the glycosyltransferase selection of these residues (Schirm et al., 2003; Thibault et al., 2001). In particular, Thibault et al. (2001) identified hydrophobic amino acid sequences preceding their identified sites of flagellin modification in *C. jejuni.* Hydrophobic amino acids are also present in the D2/D3 domain of *A. caviae* flagellins, preceding the serine residues 159 and 161, and the serine 167 and 169 residues. However, hydrophobic amino acids did not precede threonine 155, which may show that this residue is not the desired target for glycosylation and be why mutation of this residue did not impair motility. Furthermore, the double serine residues on this peptide are both separated by hydrophobic amino acids, which led to the selection of leucine 160 and isoleucine 168 for further site‐directed mutagenesis studies (L160A and I168A). Both mutations resulted in severely reduced *A. caviae* motility and only low levels of these proteins were detected in whole‐cell and supernatant samples. It is, therefore, possible that these mutated proteins are readily degraded, or less stable than their wild‐type counterparts. It is also possible that the mutations present may affect the ability of these flagellins to be secreted or affect the folding of FlaB, having an effect on flagellar assembly.

Although there is no consensus sequence for O‐linked glycosylation, it is tempting to speculate that these local regions of hydrophobicity within the D2/D3 domains of the flagellins, may guide the glycosyltransferase Maf1, to the preferred residues for modification, allowing more frequent glycosylation of these residues. As this is not a fixed consensus sequence, it indicates the modification process to be flexible, permitting other residues to also be modified, although less commonly, and resulting in a heterogeneous flagellin glycosylation pattern that we have observed.

Methylation was also detected on lysine residues on both *A. caviae* flagellins (FlaA/B). This modification did not appear to be essential for flagellar formation and motility. As the methylated peptides were also identified in their unmodified forms. This is not the first time that methylation has been identified on glycosylated bacterial flagellins, as *S. oneidensis* flagellins have also been found to be methylated on at least five lysine residues on the dominant flagellin, FlaB (Bubendorfer et al., 2013; Sun et al., 2013). The role of flagellin methylation in *S. oneidensis* is unclear; however, mutation of the putative methyltransferase did not affect bacterial motility (Sun et al., 2013). Interestingly, the recently published structure of the Maf glycosyltransferase suggests one of its three domains shares similarities with a methyl transferase (Sulzenbacher et al., 2018).

The biological role of flagellin glycosylation is still to be determined; however, the function may be unique to the individual bacterium itself. For example, the ability of *Campylobacter* species to modify their flagellins with a variety of nonulosonic acid sugars may aid the bacterium in different circumstances. The addition of pseudaminic acid is essential for *Campylobacter* flagellation; however, the presence of other sugars, such as legionaminic acid, is required for the colonization of chickens (Howard et al., 2009).

It is now becoming clear that flagellin glycosylation is more widespread than originally believed, with reports of Gram‐positive bacteria also modifying their flagella. Some can glycosylate their flagellins with nonulosonic acids, such as *Clostridium botulinum*, where it is clear that the presence of these sugars plays a role in pathogenicity, as strains isolated from infant botulism patients were more frequently found to modify their flagella with nonulosonic acids compared to strains that did not cause disease (Faulds‐Pain et al., 2014).

The nonpathogenic microorganism, *S. oneidensis*, requires glycosylation for flagellar assembly (Bubendorfer et al., 2013; Sun et al., 2013), and site‐directed mutagenesis studies on the dominant flagellin FlaB have demonstrated that each glycosylation site contributes toward flagellar function, with the modification of one site, in particular, being critical for the motility of *S. oneidensis* (Sun et al., 2013). Therefore, this modification is not only utilized by pathogenic bacteria but contributes to the bacterial colonization of a variety of environments. There are also a number of bacteria that can glycosylate their flagellins but do not require this modification for flagellar formation and motility, although it is clear that this modification aids bacterial colonization and virulence. For example, *Pseudomonas syringae*, a tobacco plant pathogen, can *O*‐glycosylate its flagellins with modified rhamnose residues (Takeuchi et al., 2003), and although this is not essential for swimming and swarming motility, glycosylation mutants have a decreased ability to adhere to surfaces and cause disease in the plant (Taguchi et al., 2006). Site‐directed mutagenesis studies have revealed that sites of modification located on the surface of the flagellum are more essential for bacterial virulence (Taguchi et al., 2006). However, the individual site‐directed mutagenesis of the known sites of modification were all found to impair motility, compared to when optimum levels of flagellin glycosylation are present (Taguchi et al., 2010). Glycosylated flagellins have been found to polymerize into a more stable flagellum, being more heat resistant than the unglycosylated equivalent (Taguchi et al., 2009). Furthermore, it is thought this stability may aid evasion of host defenses, as the host immune system (TLR‐5) recognizes an N‐terminal region of the flagellins (Andersen‐Nissen et al., 2005), so once polymerized, this N‐terminal region is hidden. The unglycosylated monomer and polymerized flagellins, and glycosylated monomer flagellins, displayed increased interactions with the host immune system, leading to increase apoptosis, compared to the glycosylated, polymerized flagellins (Taguchi et al., 2009).

It is already well established that flagella contribute toward adhesion to host cells and colonization, so it may be that glycosylated flagella allow bacteria that possess only polar flagella in this gastrointestinal environment to compete with other pathogenic microorganisms. The MS and site‐directed mutagenesis work carried out here suggest that the *A. caviae* Sch3 flagellum can be created from a variety of flagellin glycoforms, with some sites of glycosylation being preferred and occupied more frequently, suggesting partial selectivity (likely due to regions of local hydrophobicity) in Maf1 selection of modification sites. Site‐directed mutagenesis studies have suggested that optimal flagellar function may not be possible with the elimination of certain glycosylation sites, and therefore the removal of certain flagellin glycoforms. This may show that flagella are made up of a variety of flagellin glycoforms that allow efficient motility through aqueous environments and the production of favorable interactions with the environment.

## AUTHOR CONTRIBUTIONS

**Rebecca C. Lowry:** Conceptualization (equal); data curation (lead); formal analysis (lead); investigation (lead). **Laila Allihaybi:** Data curation (equal); formal analysis (equal); investigation (equal); methodology (equal). **Jennifer L. Parker:** Data curation (equal); formal analysis (equal); investigation (equal); methodology (equal). **Narciso A. S. Couto:** Data curation (equal); formal analysis (equal); investigation (equal); methodology (equal). **Graham P. Stafford:** Conceptualization (equal); data curation (equal); formal analysis (equal); funding acquisition (equal); project administration (equal); writing— review and editing (equal). **Jonathan G. Shaw:** Conceptualization (equal); data curation (equal); formal analysis (equal); project administration (equal); resources (equal); writing—original draft (lead); writing—review and editing (lead).

## CONFLICT OF INTEREST

None declared.

## ETHICS STATEMENT

None declared.

## Data Availability

All data are provided within this manuscript. Data for the manual analysis of the flagellin glycopeptides are available in the Zenodo repository at https://doi.org/10.5281/zenodo.6796498.
